# Exogenous lactate infusion (ELI) in traumatic brain injury: higher dose is better?

**DOI:** 10.1186/s13054-025-05374-y

**Published:** 2025-04-14

**Authors:** Paul Vespa, Stephanie Wolahan, Manuel Buitrago-Blanco, Courtney Real, Jesus Ruiz-Tejeda, David L. McArthur, Jeffrey N. Chiang, Denes Agoston, Thomas C. Glenn

**Affiliations:** 1 UCLA Department of Neurology, UCLA Department of Neurosurgery, UCLA Brain Injury Research Center, David Geffen School of Medicine at UCLA, 757 Westwood Blvd., Room 6236 A, Los Angeles, CA 90095 USA; 2 Department of Oncology, University of California, Irvine, Irvine, USA; 3 Uniformed Services University, Bethesda, USA

**Keywords:** Traumatic brain injury, Oxidative metabolism, Lactate, Intracranial pressure, Coma, Metabolic crisis

## Abstract

**Background/objective:**

Traumatic brain injury (TBI) is a life-threatening critical neurological injury resulting in widespread metabolic dysfunction in need of novel metabolic therapy. Exogenous lactate appears to improve brain metabolism, but the dose of lactate required remains uncertain. However, the ideal dose of lactate remains unclear. We present a comparison of low vs high dose exogenous sodium lactate infusion in a small cohort and the previous existing literature. We propose a systematic protocol to better study the question of dose–effect n in a future larger study.

**Methods:**

We analyzed the metabolic and physiologic effects of various doses of exogenous sodium lactate infusion (ELI) in the existing published literature and our own, single center cohort of patients with coma from severe TBI. Low dose ELI targeting arterial lactate concentration of 2–3 mMol was compared with high dose ELI targeting 4–6 mM. Effects of ELI on brain metabolism and intracranial pressure (ICP) were reviewed. A precision high-dose protocol was piloted and results compared against the existing literature.

**Results:**

Across various studies, metabolic response to ELI was variable and not consistently beneficial. High-dose ELI targeting arterial concentration of 4–6 mM resulted in consistent metabolic improvement and in ICP reduction (*p* < 0.01). The precision high dose protocol reliably resulted in higher arterial concentration.

**Conclusions:**

High dose ELI appears to have more consistent beneficial effects on brain metabolism and intracranial pressure.

**Trial registration:**

ClinicalTrials.gov ID NCT02776488. Date registered: 2016-05-17. Retrospectively Registered.

**Supplementary Information:**

The online version contains supplementary material available at 10.1186/s13054-025-05374-y.

## Background

Traumatic brain injury is a devastating illness with a high social and economic cost. TBI results in structural damage with immediate consequences and delayed structural and metabolic changes that can have long lasting consequences. Delayed metabolic changes are related to reduction in brain oxidative metabolism [1] which may be severe enough to result in metabolic crisis and structural secondary injury. The extent of reduction cerebral metabolic rate of oxygen correlates positively with mortality and poor functional outcome [2–4]. The reduction in oxidative metabolism is related to an underlying reduction in glucose metabolism and a general lack of available fuel to meet the increased metabolic demand of injury-induced metabolic stress [3, 4]. Human studies point to a reduction in glycolysis and resultant unavailability of the downstream metabolic products lactate which serves a fuel to brain cells [5]. Lactate is transformed to pyruvate and then Acetyl-CoA to enter the Krebs cycle and subsequently the electron transport chain to generate ATP. Lack of available pyruvate has been found in human TBI microdialysis studies, confirming a trauma-related deficiency in aerobic glycolysis [6]. Recognition of deficient aerobic glycolysis has led to experimental paradigms to seek alternative fuels such as lactate, pyruvate and ketones that can effectively bypass glycolysis to enhance brain oxidative metabolism, and ultimately improve outcome [7–11]. Animal TBI studies have shown that lactate infusion improves survival and cognitive function [7, 9]. Intraperitoneal pyruvate infusion after TBI raises serum lactate led to improved cerebral oxidative metabolism [10] and enhanced working memory after experimental TBI [11] likely by enhancing mitochondrial oxidative metabolism [12]. Exogenous lactate administration has been studied at various doses in human TBI [13–24], but the brain’s metabolic response has been variable, possibly related to underdosing of lactate. The question of whether the lactate dose matters has now been raised but there is a paucity of published data regarding whether high dose vs low dose matters. To address this question, we performed we reviewed the effects on ICP and brain metabolism of low dose vs high dose sodium lactate in the literature and our own cohort.

We reviewed data from published studies of sodium lactate infusion in TBI as well as unpublished data from our own cohort at UCLA. In these studies, a total of 178 patients with severe TBI were studied using a variety of target serum lactate concentrations ranging from 1.1 to 6.1 mMol. Patients were treated in the acute intensive care phase of illness according to standard guidelines [25]. Variable measures of brain metabolism and intracranial pressure were monitored for treatment effect including brain lactate uptake, cerebral microdialysis measures of glucose, lactate and pyruvate, and intracranial pressure [26]. Most studies used no control infusion whereas a few used hypertonic saline as a control. The duration of ELI ranged from 15 min-bolus to three-hour infusion. The studies reported no adverse effects of ELI. The studies are summarized in Table 1.

**Table 1:** Summary of existing human studies on exogenous sodium lactate infusion in TBI

Lactate infusion study	Infusion duration	Subjects n	Blood lactate mMol (sd)	Metabolic measure	Desired metabolic response
Ichai 2009	15 min	17	2.7 (0.2)	ICP	Decrease ICP by 8 mm Hg peak effect
Ichai 2013	48 h	30	1.9 (0.7)	ICP	Lower incidence of elevated ICP
Bouzat 2014	3 h	15	6.1 (1.6)	MD glucose and pyruvate	Increase in all subjects
Glenn 2015	3 h	12	1.1 (0.4)	Fractional Excretion (FE) of lactate	FE Lactate similar between controls and TBI at this concentration
Quintard 2016	3 h	25	4 (1)	MD glucose	Increase in 54% of subjects
Wolohan 2018	3 h	11	1.84 (0.5)	Brain Lactate uptake (AVDlac)	Variable increase across subjects from − 0.078 to + 0.090
Carteron 2018	3 h	23	5.1 (1.3)	MD glucose, TCD MCA and TCD PI	MD glucose + 45% TCD MCA + 21–66% TCD PI—12–26%
Bernini 2022	Bolus 20 min	17	2.9 (2.6)	MD glucose, pyruvate ICP	MD no change ICP reduction in all
Plourde 2024	Bolus 15 min	23	2.1 [IQR 1.6–3.2]	AVDlac	Increase AVDlac + 0.1 [− 0.08 to 0.2]
Current high dose cohort	3 h	5	4.73 (0.48)	Oxidative metabolism, ICP, MD glucose	Increase oxidative metabolism (decrease SOD2), reduced ICP, no change MD glucose

*MD* microdialysis, *PbtO2* brain tissue oxygen partial pressure, *AVDlac* arteriojugular difference in lactate concentration, *SOD2* superoxide dismutase 2, *ICP* intracranial pressure, *FE* fractional excretion, *TCD* transcranial doppler

**Fig. 1:**
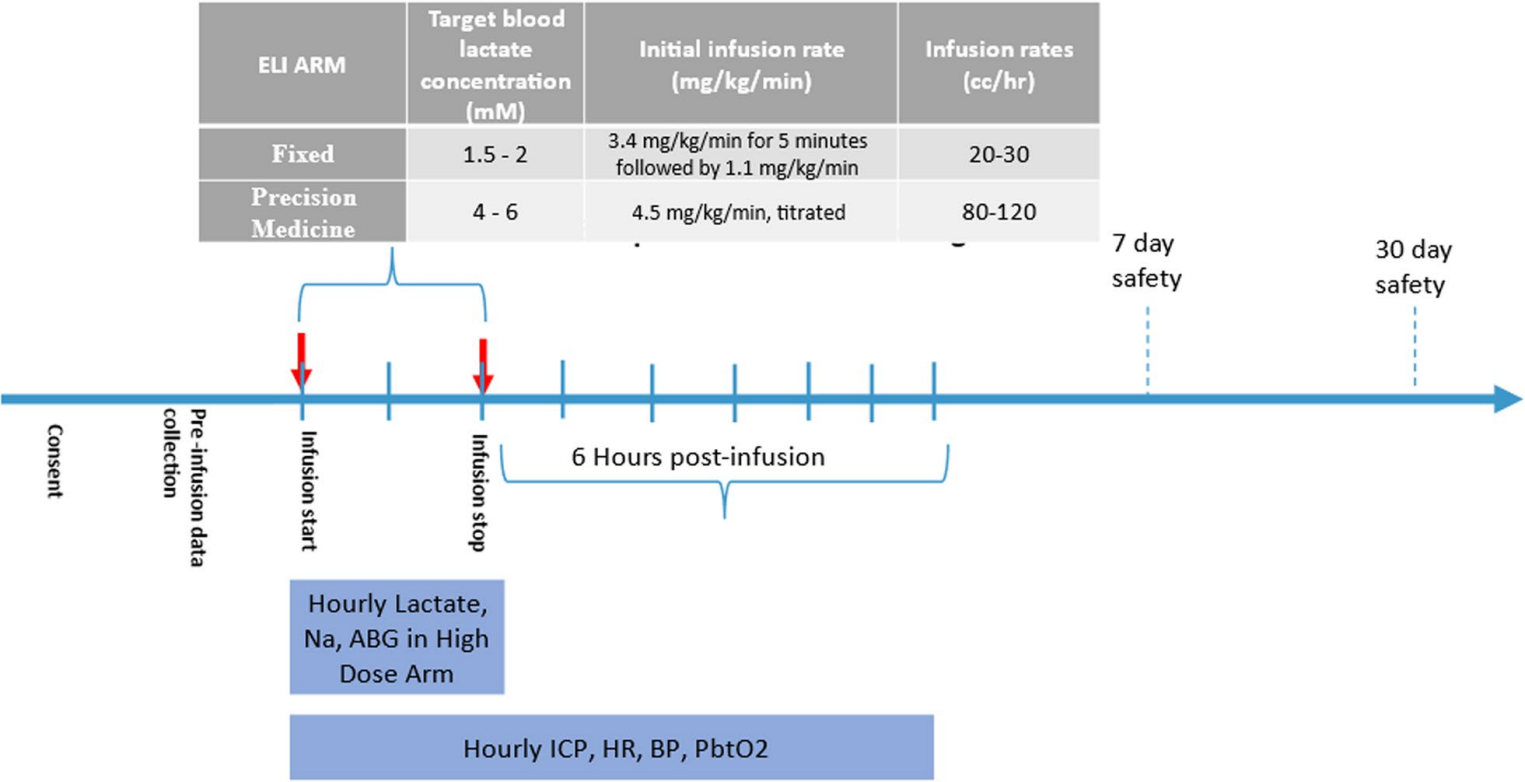
Timeline and key steps in the ELI infusion protocols used for low and high dose infusions

In our most recent unpublished cohort, we employed a novel precision high-dose lactate infusion regimen: In the precision-high dose ELI arm, precision titration of high dose sodium lactate for three hours in which the infusion was started at a calculated weight-based dose and then adjusted based on point of care measures of blood lactate. The infusion began at 4.5 mg/kg/min with precision titration to serum lactate levels using a point of care lactate meter (Nova-Point®) every 15 min targeting a desired arterial lactate concentration of 4–6 mMol. Infusion rates varied from 20 to 120 ml/hour for 3 h. A maximum of 120 ml/hr was used for those patients with weight exceeding 90 kg. Through a separate peripheral venous line, point-of-care testing was performed using 1 ml blood samples obtained at baseline and then every 15 min during the infusion. The exogenous sodium lactate infusion (ELI) was titrated by increasing or decreasing the infusion rate using a structured written protocol if blood lactate POC values were outside the desired range. Figure 1 summarizes the methods.

### Measures of oxidative metabolism

Baseline and end-of-dose serum samples were obtained in the five patients in the precision medicine ELI arm. Using the phase protein microarray (RPPM) platform, measures of brain indicators of mitochondrial oxidative metabolism, specifically, superoxide dismutase (SOD 2) and biomarkers of brain injury (Caspase 9, UCH-L1, GFAP) were assayed using 100 uL aliquots. The methods have been previously published in detail [27–30]. Within subject comparison of baseline to end-of-dose values was done.

For exploratory longitudinal statistical analyses, R package [31] and bootstrap statistics [32, 33] was used to conduct mixed models’ analysis of the change in value during the ELI with the fixed effects of infusion time and arterial lactate concentrations, or serial measures of ICP.

The review of existing studies demonstrates variability of effect of ELI on brain metabolism and intracranial pressure. There were three studies demonstrating lactate uptake and metabolism by the brain as a fuel. The manner of measuring brain uptake varies from measures of arterio-venous difference in lactate, to microdialysis measures of glucose sparing (indirectly indicating brain uptake and consumption of lactate) to isoptomer tracer studies of lactate demonstrating lactate uptake and consumption. It is difficult to compare these various methods statistically. However, the extent of uptake and metabolism by the brain has considerable variation within studies and across studies, with ranges of no effect in metabolism in some studies in which the lactate concentration was around 3 mMol and considerable effect on metabolism at concentrations from 4 to 6 mMol. In some studies, 20–60% of patients had the desired metabolic response, albeit different parameters to indicate response were used across studies. The three studies aiming for lactate concentration from 4 to 6 mMol, demonstrated the most consistency in the desired metabolic response [18, 20, and the current study]. We selected the precision target of 4–6 mMol due to the suggestion that this concentration was needed to see a consistent metabolic effect and had previously been shown to be feasible and safe in published studies.

**Fig. 2:**
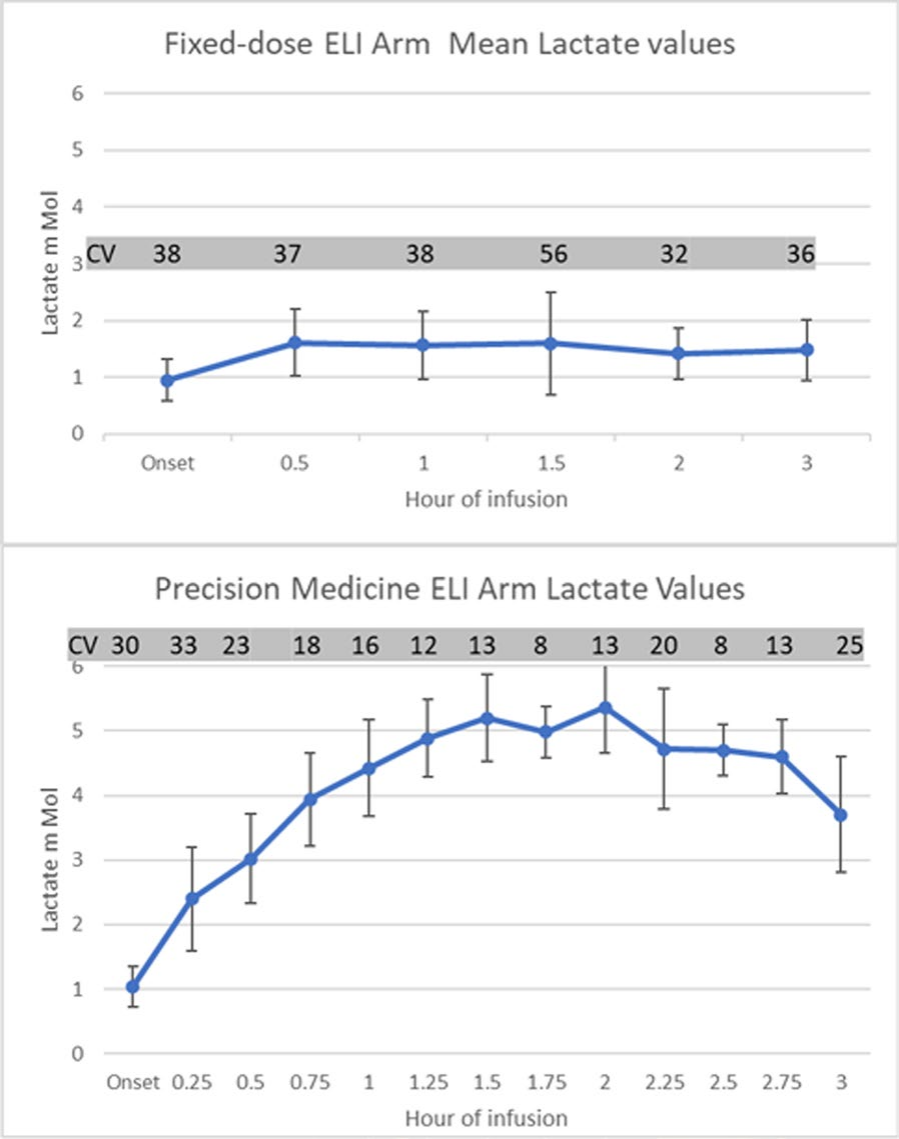
Changes in blood lactate concentration during left) fixed-low-dose ELI, with increase up to 3 times baseline, into the 2–3 mM range. On the right), precision titrated high dose infusion with increase up to 6 times baseline, into the 4–6 mM range. Grey shading indicates timing of ELI. CV = coefficient of variation (in grey box for emphasis)

At UCLA, we compared the metabolic effects of our historical fixed-dose low lactate concentration target with a novel precision-high dose lactate infusion. The differences in concentration over a 3-h infusion are shown in Fig. 2.

### Improvement in brain oxidative metabolism

As compared with the fixed-dose low concentration ELI, the UCLA precision-dose ELI resulted in improvement in brain oxidative metabolism and reduction in ICP. There was an 8% increase in mitochondrial oxidative rate, as determined by an increase in mitochondrial SOD 2, *p* < 0.01. Reduction in tissue injury markers Caspase 9 (*p* < 0.01), UCLH-1 (*p* < 0.02) but not GFAP (*p* = 0.08) occurred with high dose ELI. Cerebral microdialysis measures of metabolism were obtained in a subgroup of patients in both arms but no consistent changes in microdialysis glucose, or lactate/pyruvate ratio were seen (supplemental data).

### ICP reduction

Using a linear mixed effects modeling of ICP as a function of time (change in post-injury hour relative to infusion start), ICP decreased gradually during the infusion study at a rate of 1.2 mm Hg/hr (95% CI [0.7–1.8]) arms. ICP then returned to baseline after the infusion. The mean change in ICP was only statistically significant only in the high-dose precision medicine ELI arm (*p* < 0.001) (Supplemental data).

Safety: Safety of both the fixed-low dose and precision-high-dose infusion was assessed by monitoring for changes in blood pressure, ICP, arterial pH, venous sodium, cardiac rhythm and rate as well as evidence of pulmonary edema. Safety data are provided in the supplemental data. In this study, we demonstrated the acute physiological effects of two dosing regimens of ELI, fixed-low-dose vs. precision-high dose dosing after severe TBI. The study provides evidence that titration of high dose ELI is feasible and safe, and has positive effects on brain metabolism and ICP.

## Discussion: does the dose of lactate matter?

For the effect on brain metabolism, the answer appears to be yes. Review of the metabolic responses show inconsistent metabolic responses when the goal of ELI is low lactate concentration. The desired metabolic effect is most consistently seen with higher goal lactate concentrations, from 4.73 ± 0.48 to 6.1 ± 1.6 mMol range. The data are somewhat sparse, but the conservative preliminary conclusion is that higher dose matters.

For the effect on ICP, our recent data taken together with the studies by Ichai [18, 19] and Bernini et al. [23], suggest that lactate concentrations from 1.9 ± 0.7 to 4.73 ± 0.48 mMol are likely to reduce ICP. The effect on ICP may be due to osmolarity changes with higher dose sodium lactate, or may be indirectly due to greater metabolic changes and resulting effects on ICP.

A significant unknown variable on the effect of ELI is the baseline metabolic state of the brain prior to infusion. In those patients with metabolic crisis, as indicated by elevated microdialysis lactate/pyruvate ratio appear to have a positive metabolic response to exogenous lactate infusion [18, 19]. This metabolic effect may be dose-dependent but we have too little comparator data to know for certain. However, across all studies, a more consistent and positive effect on the desired metabolic response occurred when serum lactate concentrations are higher. In our current cohort, we found that higher arterial lactate concentration in the 4–6 mMol range provided a better metabolic response than at 2 mMol range.

Is higher dose as safe as lower dose? At this time, there have been no reports of safety concerns at either low of high dose ELI. However, mostly short duration infusions have been reported. Demonstration of safety using a higher dose of ELI, repeated dosing of ELI, or longer durations of ELI is required.

We recognize several important limitations in our knowledge regarding ELI. First, there is limited published experience with this proposed treatment, and our recent data set is also quite small. Second, the effects of ELI on ICP may be related to osmolar effects and not metabolic effects. ICP reduction may be the reason for the metabolic change through secondary effects. Third, the durability of ELI is not clear, and neither bolus administration nor three-hour infusion are likely sufficient to make a difference in outcome. We need to establish an optimal dosing and duration treatment protocol. Our current study begins to address this shortcoming. Fourth, we likely don’t understand the time window for treatment nor the proper selection criteria for treatment. Finally, we need outcomes studies to assess long term effects of ELI rather than merely biomarker studies. The existing literature does suggest that the presence of metabolic crisis may represent an indication for the use of ELI, but this remains to be demonstrated prospectively.

At this point, the use of exogenous lactate infusion in TBI appears promising, and the dose of lactate may matter. The proposed point-of-care titration protocol in this paper provides a practical method to further study the effect of dose on outcome.

## Supplementary Information

Additional file 1

## Data Availability

Availability of data: The data will be available for sharing through an institutional data use agreement administered by the UCLA Technology Development Group, https://tdg.ucla.edu/.

## References

[1] Vespa P, Bergsneider M, Hattori N, Wu HM, Huang SC, Martin NA, Glenn TC, McArthur DL, Hovda DA. Metabolic crisis without brain ischemia is common after traumatic brain injury: a combined microdialysis and positron emission tomography study. J Cereb Blood Flow Metab. 2005;25:763–74. 15716852 10.1038/sj.jcbfm.9600073 PMC4347944

[2] Xu Y, McArthur DL, Alger JR, Etchepare M, Hovda DA, Glenn TC, Huang S, Dinov I, Vespa PM. Early nonischemic oxidative metabolic dysfunction leads to chronic brain atrophy in traumatic brain injury. J Cereb Blood Flow Metab. 2010;30:883–94. 20029449 10.1038/jcbfm.2009.263 PMC2949156

[3] Jaggi JL, Obrist WD, Gennarelli TA, Langfit TW. Relationship of early cerebral blood flow and metabolism to outcome in acute head injury. J Neurosurg. 1990;72:176–82. 10.3171/jns.1990.72.2.0176. 2295915 10.3171/jns.1990.72.2.0176

[4] Glenn TC, Kelly DF, Boscardin WJ, McArthur DL, Vespa PM, Oertel M, Hovda DA, Bergsneider M, Hillered L, Martin NA. Energy dysfunction as a predictor of outcome after moderate or severe head injury: Indices of oxygen, glucose and lactate metabolism. J Cereb Blood Flow Metab. 2003;23:1239–50. 14526234 10.1097/01.WCB.0000089833.23606.7F

[5] Pellerin L, Magistretti PJ. Sweet sixteen for ANLS. J Cereb Blood Flow Metab. 2012;32:1152–66. 22027938 10.1038/jcbfm.2011.149 PMC3390819

[6] Hillered L, Vespa PM, Hovda DA. Translational neurochemical research in acute human brain injury: the current status and potential future for cerebral microdialysis. J Neurotrauma. 2005;2:3–41. 10.1089/neu.2005.22.3 15665601

[7] Rice AC, Zsoldos R, Chen T, Wilson MS, Alessandri B, Hamm RJ, Bullock MR. Lactate administration attenuates cognitive deficits following traumatic brain injury. Brain Res. 2002;928(1–2):156–9. 11844482 10.1016/s0006-8993(01)03299-1

[8] Chen T, Qian YZ, Rice A, Zhu JP, Di X, Bullock R. Brain lactate uptake increases at the site of impact after traumatic brain injury. Brain Res. 2000;86:281–7. 10.1016/s0006-8993(00)01992-2 10760489

[9] Holloway R, Zhou Z, Harvey HB, Levasseur JE, Rice AC, Sun D, Hamm RJ, Bullock MR. Effect of lactate therapy upon cognitive deficits after traumatic brain injury in the rat. Acta Neurochir. 2007;149:919–27. 17660938 10.1007/s00701-007-1241-y

[10] Moro N, Ghavim SS, Harris NG, Hovda DA, Sutton RL. Pyruvate treatment attenuates cerebral metabolic depression and neuronal loss after experimental traumatic brain injury. Brain Res. 2016;1642:270–7. 27059390 10.1016/j.brainres.2016.04.005 PMC4899222

[11] Moro N, Ghavim SS, Hovda DA, Sutton RL. Delayed sodium pyruvate treatment improves working memory following experimental traumatic brain injury. Neurosci Lett. 2011;491(2):158–62. 21241774 10.1016/j.neulet.2011.01.029 PMC3045674

[12] Greco T, Vespa P, Prins M. Alternative substrate metabolism depends on metabolic state after TBI. Exp Neurol. 2020;329:113289. 32247790 10.1016/j.expneurol.2020.113289 PMC8168752

[13] Glenn TC, Martin NA, Horning MA, McArthur DL, Hovda DA, Vespa P, Brooks GA. Lactate: brain fuel in human traumatic brain injury: a comparison with normal healthy control patients. J Neurotrauma. 2015;32:820–32. 25594628 10.1089/neu.2014.3483 PMC4530406

[14] Wolahan SM, Mao HC, Real C, Vespa PM, Glenn TC. Lactate supplementation in severe traumatic brain injured adults by primed constant infusion of sodium L-lactate. J Neurosci Res. 2017;96:688. 28543565 10.1002/jnr.24085 PMC5696121

[15] Emhoff CA, Messonnier LA, Horning MA, Fattor JA, Carlson TJ, Brooks GA. Direct and indirect lactate oxidation in trained and untrained men. J Appl Physiol. 2013;115:829–38. 23788576 10.1152/japplphysiol.00538.2013 PMC8846964

[16] Wolahan SM, Mao HC, Real C, Vespa PM, Glenn TC. Lactate supplementation in severe traumatic brain injured adults by primed constant infusion of sodium L-lactate. J Neurosci Res. 2018;96:688–95. 28543565 10.1002/jnr.24085 PMC5696121

[17] Bisri T, Utomo BA, Fuadi I. Exogenous lactate infusion improved neurocognitive function of patients with mild traumatic brain injury. Asian J Neurosurg. 2016;11(2):151–9. 27057222 10.4103/1793-5482.145375 PMC4802937

[18] Quintard H, Patet C, Zerlauth JB, Suys T, Bouzat P, Pellerin L, Meuli R, Magistretti PJ, Oddo M. Improvement of neuroenergetics by hypertonic lactate therapy in patients with traumatic brain injury is dependent on baseline cerebral lactate/pyruvate ratio. J Neurotrauma. 2016;33(7):681–7. 26421521 10.1089/neu.2015.4057 PMC4827289

[19] Bouzat P, Sala N, Suys T, Zerlauth J-B, Marques-Vidal P, Feihl F, Bloch J, et al. Cerebral metabolic effects of exogenous lactate supplementation on the injured human brain. Intensive Care Med. 2014;40:412–21. 24477453 10.1007/s00134-013-3203-6

[20] Ichai C, Armando G, Orban JC, Berthier F, Rami L, Samat-Long C, Grimaud D, Leverve X. Sodium lactate versus mannitol in the treatment of intracranial hypertensive episodes in severe traumatic brain-injured patients. Intensive Care Med. 2009;35:471–9. 18807008 10.1007/s00134-008-1283-5

[21] Ichai C, Payen J-F, Orban J-C, Quintard H, Roth H, Legrand R, Francony G, Leverve XM. Half-molar sodium lactate infusion to prevent intracranial hypertensive episodes in severe traumatic brain injured patients: a randomized controlled trial. Intens Care Med. 2013;39:1413–22. 10.1007/s00134-013-2978-9 23749153

[22] Carteron L, Solari D, Patet C, Quintard H, Miroz JP, Bloch J, Daniel RT, Hirt L, Eckert P, Magistretti PJ, Oddo M. Hypertonic lactate to improve cerebral perfusion and glucose availability after acute brain injury. Crit Care Med. 2018;46(10):1649–55. 29923931 10.1097/CCM.0000000000003274

[23] Plourde G, Ichai C, Quintard H. Cerebral lactate uptake after half-molar sodium lactate therapy in traumatic brain injury: a brief report. J Neurotrauma. 2024. 10.1089/neu.2023.0508. 38420880 10.1089/neu.2023.0508

[24] Bernini A, Miroz JP, Abed-Maillard S, Favre E, Iaquaniello C, Ben-Hamouda N, Oddo M. Hypertonic lactate for the treatment of intracranial hypertension in patients with acute brain injury. Sci Rep. 2022;12(1):3035. 10.1038/s41598-022-07129-z. 35194150 10.1038/s41598-022-07129-z PMC8864009

[25] Brain Trauma Foundation; American Association of Neurological Surgeons; Congress of Neurological Surgeons. Guidelines for the management of severe traumatic brain injury. J Neurotrauma. 2007. 10.1089/neu.2007.9999. 10.1089/neu.2007.9999 17511534

[26] Vespa P, Tubi M, Claassen J, Blanco M, McArthur D, Velazquez AG, Tu B, Prins M, Nuwer M. Metabolic crisis occurs with seizures and periodic discharges after brain trauma. Ann Neurol. 2016;79:579–90. 10.1002/ana.24606. 26814699 10.1002/ana.24606

[27] Paweletz CP, et al. Reverse phase protein microarrays which capture disease progression show activation of pro-survival pathways at the cancer invasion front. Oncogene. 2001;20(16):1981–9. 11360182 10.1038/sj.onc.1204265

[28] Pierobon M, et al. Reverse-phase protein microarrays. Methods Mol Biol. 2012;823:215–35. 22081348 10.1007/978-1-60327-216-2_14

[29] Agoston DV, Elsayed M. Serum-based protein biomarkers in blast-induced traumatic brain injury spectrum disorder. Front Neurol. 2012;3:107. 22783223 10.3389/fneur.2012.00107 PMC3390892

[30] Agoston DV, Shutes-David A, Peskind ER. Biofluid biomarkers of traumatic brain injury. Brain Inj. 2017;31(9):1195–203. 28981341 10.1080/02699052.2017.1357836

[31] Pinheiro JC, Bates DM. Mixed-effects models in S and S-PLUS_. Springer, New York. 2000. 10.1007/b98882.

[32] Wilcox RR. Introduction to robust estimation and hypothesis testing. 5th ed. London: Elsevier; 2022.

[33] Efron B. Bootstrap methods: another look at the Jackknife. Ann Statist. 1979;7(1):1–26. 10.1214/aos/1176344552.

