# Supplementary material for: Exogenous lactate infusion (ELI) in traumatic brain injury: higher dose is better?
Source: Crit Care. 2025 Apr 14;29:153. doi: 10.1186/s13054-025-05374-y (PMC11998250; doi:10.1186/s13054-025-05374-y)
Supplement: Supplementary file 1 — Additional file 1 [file 13054_2025_5374_MOESM1_ESM.docx]

Supplemental Data – Vespa et al – ELI in TBI for Critical Care Perspectives Article 3-7-25

Legend for Supplemental Figures.

Table S1: Table comparing baseline with end infusion hour 3 values for sodium (Na), arterial pH, systolic blood pressure (SBP), and systemic oxygenation/pulmonary compromise.

Table S2: Metabolic studies obtained during precision-high-dose ELI. Two sided student’s t test values between group baseline and hour 3 sample values are shown.

Figure S1: Heart rate (HR) comparison between ELI: No overall change in HR during fixed-dose and precision medicine ELI. Hours shown are from 1 hour before ELI (-1) to 4 hours after the start of ELI infusion. PIH = post infusion hour.

Figure S2: Comparison of changes in intracranial pressure (ICP) during infusion of fixed-low-dose ELI and precision-high-dose ELI. Shaded gray area reflects hours of infusion.

Figure S3: Mean hourly cerebral microdialysis (MD) glucose values from post-injury hour 11 through post-injury hour 190 (with standard deviations = SD) in 10 patients. Summary values are: mean glucose (mean 1.21 ± 0.31 mM, IQR 0.95-1.39, 0.44)

Figure S4: Mean hourly cerebral microdialysis (MD) LPR values from post-injury hour 11 through post-injury hour 190 (with standard deviations = SD) in 10 patients. Summary values are: (mean 32.3 ± 8.7, IQR 28.2-34.6, 6.3)

Figure S5: Mean hourly cerebral microdialysis (MD) pyruvate values from post-injury hour 11 through post-injury hour 190 (with standard deviations = SD) in 10 patients. Summary values are: mean 172 ± 37.3 uM, IQR 148.5-193.2, 44.7).

Figure S6: Hourly values of cerebral microdialysis (MD) values before, during and after the fixed-dose ELI, n=6 subjects. Blue shading indicates time of ELI.

Figure S7: Hourly values of cerebral microdialysis (MD) values before, during and after the precision medicine ELI, n= 3 subjects. Blue shading indicated time of ELI. A fourth patient in the high ELI dose had completion of microdialysis prior to ELI, and these data are not included in this graph.

Supplemental Table 1: Safety Data for fixed-low-dose and precision-high-dose ELI

| **Sodium** | **N** | **Avg [Na] before** | **Avg [Na] after** | **Δ[Na]** | **Δ[Na] 95% CI** | **p-value** |
| --- | --- | --- | --- | --- | --- | --- |
| Precision | **5** | **147.7** | **159.0** | **11.3** | **[6.1 16.5]** | **0.011** |
| Fixed | 11 | 144.9 | 147.7 | 2.8 | [0.0 5.6] | 0.052 |
| **pH** |  |  |  |  |  |  |
| Precision | **5** | **7.43** | **7.47** | **0.037** | **[-0.121 0.048]** | **0.203** |
| Fixed | 11 | 7.43 | 7.44 | 0.014 | [-0.042 0.013] | 0.249 |
| **SBP** |  |  |  |  |  |  |
| Precision | **5** | **135.4** | **139.7** | **4.3** | **[-14.2 22.8]** | **0.43** |
| Fixed | 11 | 139.7 | 138.5 | -1.2 | [-11.6 9.3] | 0.79 |
| **Pulmonary insufficiency** |  | **Hypoxemia SaO2 < 92% before** | **Hypoxemia SaO2 < 92% after** |  |  |  |
| Precision | 5 | 0 | 0 |  |  | NA |
| Fixed | 11 | 0 | 0 |  |  | NA |

Supplemental Table 2: Metabolic biomarkers obtained during precision-high-dose ELI.

| **Biomarker** | |  | **Change after 3 hours of Precision Medicine ELI** | **P value** | **Interpretation** |
| --- | --- | --- | --- | --- | --- |
| SOD2 | Superoxide dismutase-2, mitochondrial |  | Decreased by 8.5% | 0.0058 | An increase in oxidative metabolism/mitochondrial respiration transiently during the 3 hour ELI |
| GFAP | Glial fibrillary acidic protein |  | Decreased by 1.2% | 0.078 | Astroglial cell injury reduced |
| UCH-L1 | Ubiquitin C-terminal hydrolase L1 |  | Decreased by 5.1% | 0.019 | Neuronal cell injury reduced |
| Casp9 | Caspase 9 |  | Decreased by 7.1 % | 0.002 | Reduced apoptosis |

Supplemental Figure S1: Heart rate before and during ELI infusion


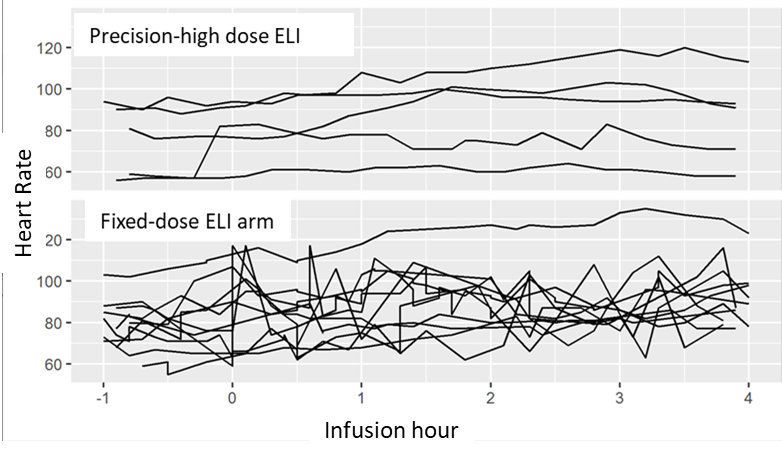


Supplemental Figure S2: Changes in intracranial pressure (ICP) during the fixed-low-dose and precision-high-dose ELI.


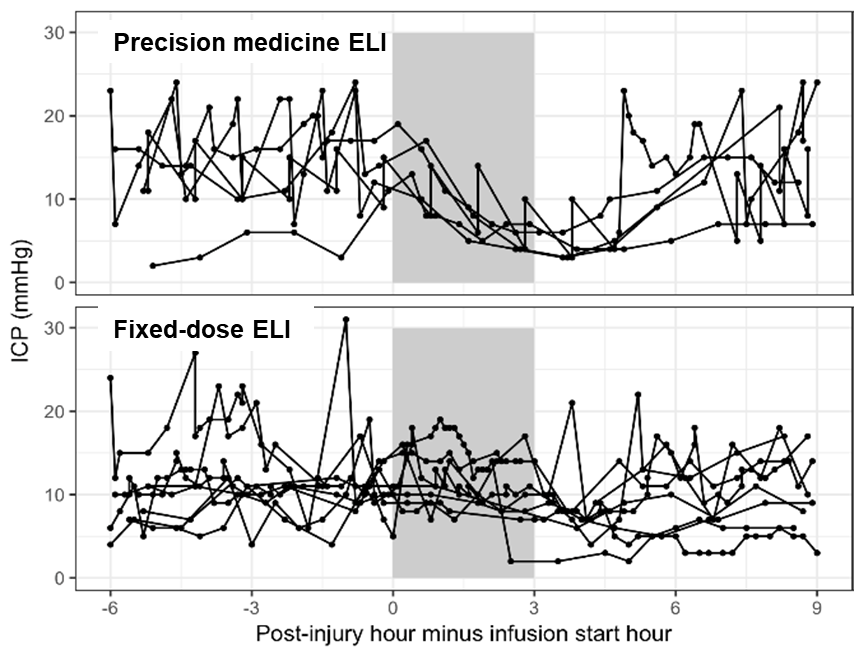


Supplemental Figure S3: Hourly mean microdialysis glucose values (mM) in 10 patients hours


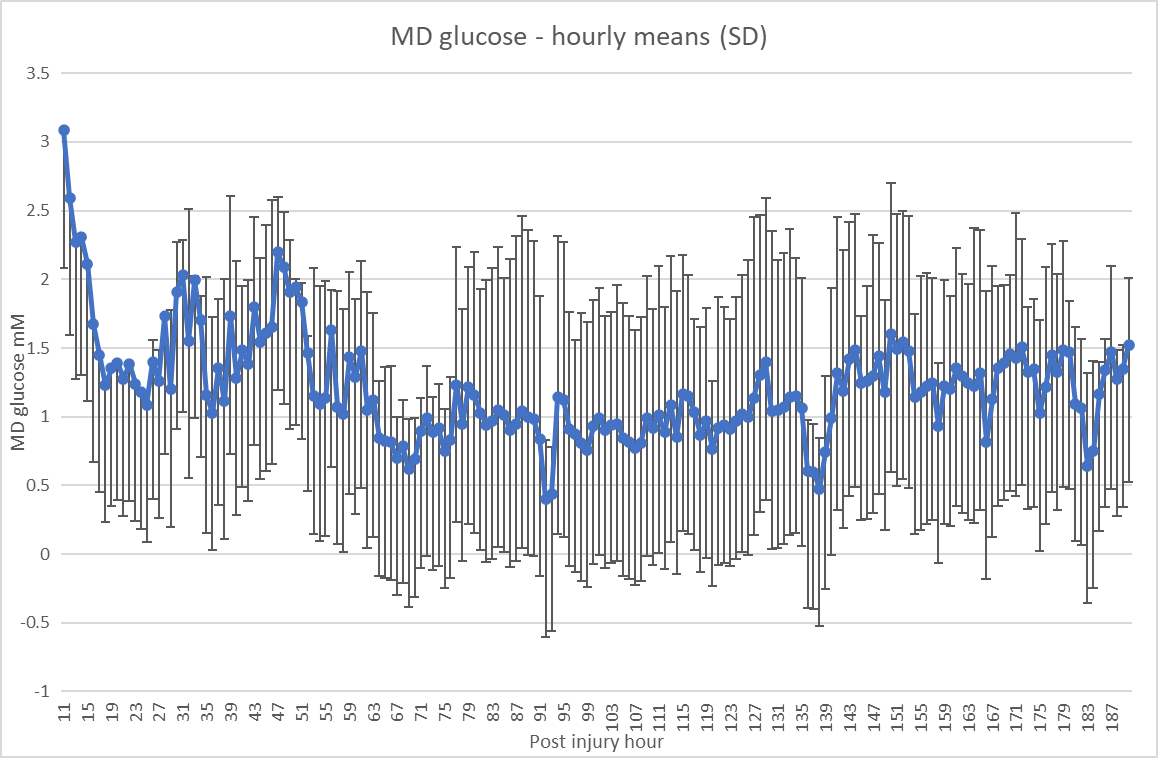


Supplemental Figure S4: Hourly mean microdialysis lactate/pyruvate values in 10 patients


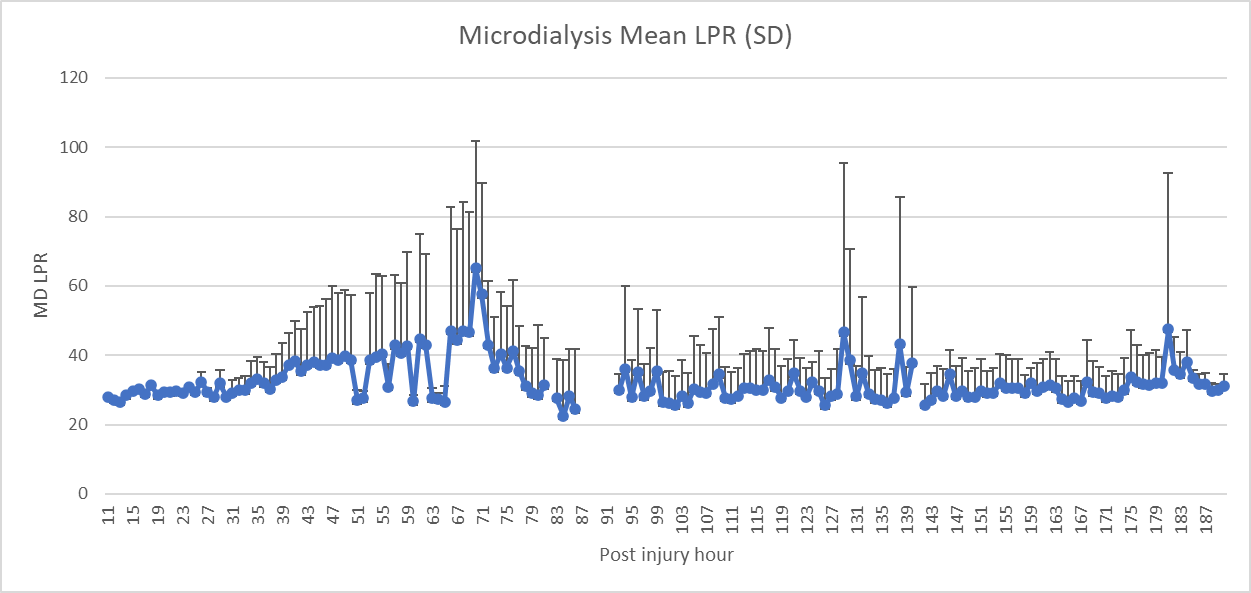


Supplemental Figure S5: Hourly mean microdialysis pyruvate values in 10 patients


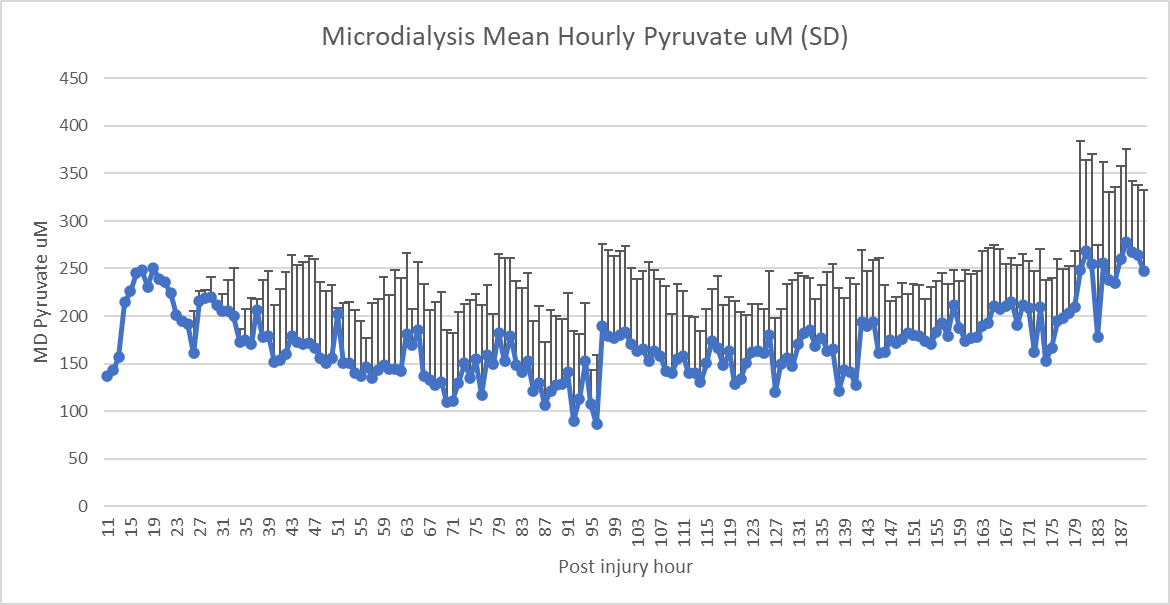


Supplemental Figure S6: Cerebral microdialysis values of during Fixed-dose ELI arm

Supplemental Figure S7: Cerebral microdialysis during precision medicine ELI arm
